# Effect of low-level laser therapy on the modulation of the mitochondrial activity of macrophages

**DOI:** 10.1590/bjpt-rbf.2014.0046

**Published:** 2014

**Authors:** Nadhia H. C. Souza, Raquel A. M. Ferrari, Daniela F. T. Silva, Fabio D. Nunes, Sandra K. Bussadori, Kristianne P. S. Fernandes

**Affiliations:** 1 Programa de Pós-graduação em Ciências da Reabilitação, Universidade Nove de Julho (UNINOVE), São Paulo, SP, Brazil; 2 Programa de Pós-graduação em Biofotônica Aplicada às Ciências da Saúde, UNINOVE, São Paulo, SP, Brazil; 3 Departamento de Ciências Exatas, UNINOVE, São Paulo, SP, Brazil; 4 Departamento de Estomatologia, Faculdade de Odontologia, Universidade de São Paulo (USP), São Paulo, SP, Brazil

**Keywords:** macrophages, low-level laser therapy, muscle repair, rehabilitation

## Abstract

**BACKGROUND:**

Macrophages play a major role among the inflammatory cells that invade muscle tissue following an injury. Low-level laser therapy (LLLT) has long been used in clinical practice to accelerate the muscle repair process. However, little is known regarding its effect on macrophages.

**OBJECTIVE:**

This study evaluated the effect of LLLT on the mitochondrial activity (MA) of macrophages.

**METHOD:**

J774 macrophages were treated with lipopolysaccharide (LPS) and interferon - gamma (IFN-γ) (activation) for 24 h to simulate an inflammatory process, then irradiated with LLLT using two sets of parameters (780 nm; 70 mW; 3 J/cm^2^ and 660 nm; 15 mW; 7.5 J/cm^2)^. Non-activated/non-irradiated cells composed the control group. MA was evaluated by the cell mitochondrial activity (MTT) assay (after 1, 3 and 5 days) in three independent experiments. The data were analyzed statistically.

**RESULTS:**

After 1 day of culture, activated and 780 nm irradiated macrophages showed lower MA than activated macrophages, but activated and 660 nm irradiated macrophages showed MA similar to activated cells. After 3 days, activated and irradiated (660 nm and 780 nm) macrophages showed greater MA than activated macrophages, and after 5 days, the activated and irradiated (660 nm and 780 nm) macrophages showed similar MA to the activated macrophages.

**CONCLUSIONS:**

These results show that 660 nm and 780 nm LLLT can modulate the cellular activation status of macrophages in inflammation, highlighting the importance of this resource and of the correct determination of its parameters in the repair process of skeletal muscle.

## Introduction

Although the pattern of gene expression in muscle regeneration parallels the pattern corresponding to embryonic muscle development, the microenvironments in which the two processes occur are dramatically different^1^.

This difference is due to the abundance of inflammatory cells in regenerative muscle, the concentration of which might exceed 100,000 cells/mm^3^^[^ 1 ^,^ 2 ^]^. These inflammatory cells are activated cells able to release numerous soluble molecules, particularly cytokines, which can affect the viability, differentiation and transcriptional activities of regenerative muscle cells^1^.

Skeletal muscle initially responds to injury through a Th1-driven inflammatory response, which mostly involves neutrophils and macrophages with the M1 phenotype. M1 macrophages release cytokines (tumor necrosis factor-alpha - TNF-α - and interleukin 6 - IL6) and proinflammatory enzymes (cyclooxygenase 2 - COX-2) and produce nitric oxide (NO); all these factors contribute to further tissue damage^1^^,^ 3 ^-^ 5.

Forty-eight hours after injury, the muscle tissue exhibits M2 macrophages, which reduce the population of M1 macrophages through the release of anti-inflammatory cytokines, including IL-10^1^. The number of M2 macrophages reaches its peak four days later and remains high for many days^1^.

The shift in macrophage phenotype from M1 to M2 is a key event in muscle regeneration and coincides with the shift from the proliferative to the early differentiation stage of myogenesis^1^.

M2 macrophages are primarily activated by cytokines IL-4, IL-10 and IL-13^6^ and express cytokines such as IL-10^1^.

The complexity and the antagonism of the macrophages phenotypes involved in the inflammatory process triggered by muscle injury point to the need to consider such cells as targets for therapeutic interventions^1^.

Among the therapeutic interventions applied to accelerate skeletal muscle repair following different types of injuries, low-level laser therapy (LLLT) stands out^7^^-^ 12.

Notwithstanding, few studies have assessed the isolated effect of LLLT on macrophages, particularly on mitochondrial activity (MA) in these cells^13^.

Based on the information above, it seems safe to assume that much research is still needed to understand the effects of laser therapy on the macrophages involved in muscle repair and to establish ideal dosimetry parameters to modulate and accelerate the process.

This study sought to contribute to filling that gap by assessing the effect of LLLT on the mitochondrial activity of (M1) macrophages activated to simulate inflammation.

## Method

### Cell culture

The macrophage J774 cell line was grown in Dulbecco's Modified Eagle Medium (DMEM, Vitrocell, Campinas, SP, Brazil) supplemented with 10% fetal bovine serum (FBS) and 2 mM L-glutamine (Vitrocell, Campinas, SP, Brazil). Cultures were kept in an incubator (HEPA *class 3110, Thermo Electron Corporation*, Marietta, OH, USA) at 37°C and in a wet environment with 5% CO_2_. Cell growth was assessed every 24 hours using an inverted phase microscope (Eclipse TE 2000U, Nikon, Melville, NY, USA).

### Inflammation simulation

Macrophages were treated with 1 µg/mL Escherichia coli (E coli) O26:B6 lipopolysaccharide (LPS) (Sigma, St. Louis, MO) and 0.2 µg/mL interferon-gamma (IFN-γ) (Sigma, St. Louis, MO, EUA) to simulate phenotype M1. To simulate inflammation and cell suffering, macrophages cells were grown in DMEM with 5% FBS^14^^-^ 17. The procedure to culture cells for the control groups was the same, but without the addition of LPS and IFN-γ. After 24 hours, the plates were washed three times with buffered saline solution. The cells were then detached using a cell scraper and transferred to 50-mL Falcon tubes (*Techno Plastic Products* [TPP], Trasadingen, Switzerland).

### Low-level laser therapy (LLLT)

The 50-mL tubes containing cell suspensions were centrifuged (1,200 rpm at 10°C for five minutes using a Centrifuge Excelsa 4-280R, Fanem, São Paulo, SP, Brazil). Then, the lower end of the tubes was subjected to irradiation from underneath, allowing the laser beam to strike the cell *pellet* without passing through the culture medium^17^. Irradiation was performed in continuous mode using a *Twin-laser (MM Optics*, São Carlos, SP, Brazil) in a partially darkened room to avoid interference from external light sources. The cells in the control group were subjected to the same procedures but were not irradiated. The irradiation parameters (described in Table 1) were selected based on previous studies^18^^-^ 20. The device output power was assessed using a power meter (*Laser Check, MM Optics*, São Carlos, SP, Brazil). Table 1 describes the output values as well as the effective values considering the passage of light through the polypropylene tubes with the cell precipitates, as previously described^21^.

**Table 1:** Low-level laser therapy (LLLT) parameters.

Wavelength (nm)	Power (mW)	Fluence (J/cm2)	Effective power (mW)	Beam spot area (cm2)	Time (s)	Irradiated area (cm2)	Effective power density (mW/cm2)	Effective fluence (J/cm2)
780	70	3	53.9	0.04	1.5 (2x)	0.196	275	0.41
660	15	7.5	11.25	0.04	20	0.196	57.4	1.15

## Experimental groups

### Group 1

Control (non-activated, non-irradiated macrophages); Group 2: macrophages activated by means of LPS and IFN-γ; Group 3: macrophages irradiated with 660-nm laser; Group 4: macrophages activated by means of LPS and IFN- γ and irradiated with 660-nm laser; Group 5: macrophages irradiated with 780-nm laser; Group 6: macrophages activated by means of LPS and IFN-γ and irradiated with 780-nm laser.

### Cell mitochondrial activity assay - MTT

The MTT assay is a colorimetric assay able to assess the ability of mitochondrial enzyme succinate dehydrogenase in viable cells to cleave MTT [3-(4,5-dimethylthiazol-2-yl)-2,5-diphnyltetrazolium bromide] tetrazolium rings, resulting in dark-blue formazan crystals. As the cell membrane is impermeable to formazan crystals, they are retained within viable cells and released following cell lysis. Macrophages (1 x 10^3^/well) were incubated in 96-well flat-bottom culture plates (TPP) with DMEM and 5% FBS for one, three and five days. They were then washed with 100 µL of phosphate-buffered saline (PBS); MTT (0.5 µg/mL) was added; and the plates were incubated three hours in a CO_2_ incubator at 37°C. Next, 100 µL of isopropanol was added, and an absorbance reading was performed at 620 nm using a plate reader (2020, *Anthos*, Eugendorf, Austria).

### Statistical analysis

All the tests were independently performed three times, and eight replicates were prepared from each sample. Data analysis included the calculation of the mean and standard deviation and analysis of variance (ANOVA), for which purpose the software *GraphPad InStat-3* was used. Statistical significance was assessed by means of Tukey's test and was considered acceptable when p≤0.05.

## Results

### Activated cells (simulated inflammation)

**Figure 1:**
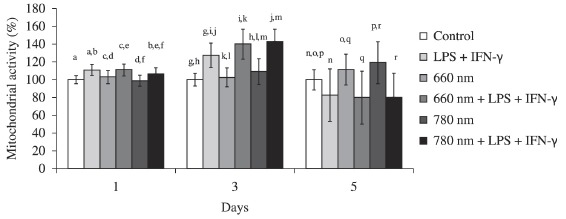
Percentage of mitochondrial activity (MTT method) in cells from the different experimental groups compared to the control group cells. The same letters represent statistically significant differences (a, c, f, g, h, j, k, m, p, q, r=p< 0.001; d, i, n=p<0.01; b, e, l, o=p<0.05).

After one day of culture, the MA of activated macrophages was similar to the MA of activated macrophages irradiated at 660 nm and higher than the MA of activated macrophages irradiated at 780 nm (p<0.05). The MA of activated macrophages irradiated at 660 nm was higher (p<0.05) than the MA of activated macrophages irradiated at 780 nm. The MA of activated macrophages was higher (p<0.001) than the MA of the control group. The MA of activated and irradiated (660 nm and 780 nm) macrophages was higher (p<0.001) than the MA of the non-activated cells irradiated with the corresponding energy parameters (Figure 1). After three days of culture, the MA of the activated macrophages irradiated at 660 nm or 780 nm laser was higher (p<0.01 and p<0.001, respectively) than the MA of the activated macrophages. The MA of the activated macrophages irradiated by the 660 nm laser was similar to the MA of the activated cells irradiated at 780 nm. The MA of the activated macrophages was not only higher (p<0.001) than the control group (non-activated, non-irradiated cells), but the difference was more patent (Figure 1).

After five days of culture, the MA of the activated macrophages was similar to the MA of activated macrophages irradiated at 660 nm or 780 nm laser. The MA of the activated macrophages irradiated at 660 nm laser was similar to the MA of the activated cells irradiated by the 780 nm laser. The MA of the activated macrophages was lower (p<0.01) than the MA of the control group (Figure 1). Upon comparison of activated and irradiated cultures (using 660- and 780-nm lasers) with irradiated non-activated cultures, an inversion of the behavior found on days one and three was observed, as the MA of the activated and irradiated cells was lower than the MA of the non-activated macrophages irradiated with the corresponding energy parameters (p<0.001).

### Non-activated cells

After one day of culture, there was no difference between the MA of the control group and the groups of irradiated cells. The MA of the cells irradiated at 660 nm was higher (p<0.01) than the MA of the cells irradiated at 780 nm (Figure 1). After three days of culture, irradiation at 660 nm did not induce changes in MA compared to non-irradiated cells, but irradiation at 780 nm induced an increase in MA compared to the control group (p<0.001). The MA of the cells irradiated at 660 nm was lower (p<0.05) than the MA of the cells irradiated at 780 nm (Figure 1). After five days of culture, irradiation at 660 nm (p<0.05) and particularly at 780 nm (p<0.001) increased the MA of the irradiated cells compared to the non-irradiated cells. The MA of cells irradiated at 660 nm was similar to the MA of cells irradiated at 780 nm (Figure 1).

## Discussion

The modulation of the various stages of skeletal muscle repair is mainly accomplished through changes in the activation profile of macrophages, resulting in changes in the phenotype and function of such cells^1^. For that reason, macrophages are considered to be targets for therapeutic intervention^1^.

Several experimental and clinical studies conducted within the context of muscle injury rehabilitation have shown that LLLT can modulate the process of muscle repair^22^^-^ 28. However, no study has yet assessed whether laser therapy can change the state of activation of macrophages.

In this study, we assessed the effect of LLLT applied with two different energy parameters on the mitochondrial activity of J774 macrophages one, three and five days after irradiation. The cells were cultured under conditions of nutrient deficiency and treated with LPS and IFN-γ to simulate inflammation and induce the appearance of macrophage phenotype M1.

Previous studies that assessed the effects of LLLT and LED (*light emitting diode*) therapy on macrophages or their precursors (monocytes) did not evaluate their mitochondrial activity but did evaluate various functions of such cells^13^^,^ 14 ^,^ 18 ^-^ 20 ^,^ 29 ^-^ 31.

Mitochondria exert a crucial modulatory effect on the pathway of activation of inflammatory macrophages that leads to the production of cytokines, i.e., the MAPK (*Mitogen Activated Protein Kinases*) and NF-κβ (Nuclear Factor-KappaB) pathways^32^. When an inflammatory stimulus (e.g., LPS + IFNγ) triggers macrophage activation, the mitochondria amplify the MAPK pathway, resulting in increases of cytokines and other inflammatory mediators production^33^. The MTT assay, which was used in this study, assesses mitochondrial activity and directly reflects the status of cell activation^32^^,^ 33.

After one and three days of culture, the MA of the macrophages treated with IFNγ and LPS increased compared to the non-activated cells, showing that the activation model used in this study was effective. The findings after five days of culture were the opposite of the earlier ones, as the MA of the activated cells was lower than in the control group. The reason might be that by 5 days, the cell activation and/or viability had decreased as a function of the intense stimulation to which they had been subjected on the previous days and/or the action of the products they secreted.

The MA of the activated cells irradiated at 780 nm decreased after one day of culture. After three days of culture, irradiation at 660 nm and 780 nm exerted a positive modulation of the MA of macrophages, which might denote an increase in cell activation. After five days of culture, irradiation with either laser no longer modulated the mitochondrial activity of the activated cells.

Relative to the non-activated cells, irradiation at 780 nm exerted a positive modulation of the MA after three days of culture. The same effect was found after five days of culture with both laser energy parameters (660 and 780 nm).

Only the study by Young et al.^13^ assessed viability and proliferation in an irradiated monocyte line, though with an 820 nm pulsed laser (15 mW; 2.4 J/cm^2^; 0.3 J). After 36 hours of culture, the *trypan* blue exclusion test showed an increase in the number of viable cells compared to the non-irradiated group. It is difficult, however, to compare those results with ours because the dosimetry parameters, methods and outcome are all different. In addition, Young et al.^13^ used monocytes, whereas we assessed a macrophage cell line.

In fact, many studies have shown that LLLT has effects on several cell types, mostly through the activation of the mitochondrial respiratory chain, resulting in increased ATP production and the induction of transcription factors^34^^,^ 35. Our findings, therefore, might indicate that the energy applied to cells by means of laser irradiation was able to stimulate the above mentioned mechanisms, increasing the activation of non-activated macrophages (780-nm laser on day three and 660- and 780-nm lasers on day five) and amplifying the effects in the activated macrophages.

Our results showed that the MA of activated cells irradiated at 780 nm was reduced after one day of culture. This finding corroborates the reports by Sousa et al.^18^, who observed a reduction in TNF-α production 24 hours after the irradiation of activated M1 macrophages using the same dosimetry parameters and methods that we used.

Although *in vitro* studies afford standardized, highly reproducible models and allow cell and molecular assessment, the results of such studies cannot be correlated with eventual clinical outcomes. Nevertheless, previous knowledge of the effect of LLLT and other therapeutic resources on the various cell types that compose muscle tissue is of paramount importance for the formulation of *in vivo* protocols that can exert more effective modulation of the muscle repair process.

In addition, accurate knowledge of the optical properties of the cells/tissues to be irradiated as well as of the barriers through which the light will pass is crucial. In the experimental model used in this study, the lasers had to pass through the test tube bottom to reach the macrophages. As a consequence, a part of the output energy was lost due to reflection, dispersion and absorption by the polypropylene that composed the test tubes^21^. For that reason, the effective (remaining) power values were included in the calculation of the power density and the energy density, which was performed according Silva et al.^21^.

For dosimetry parameters to be transferred from one experimental model to another, the behavior of light relative to the various barriers through which it passed before reaching the target should be accurately known. The absorption coefficient of the targeted tissue should also be known, as the therapeutic effects of light are fully dependent on the amount of absorbed energy.

Once the appropriate experimental data at the cell level and the data from animal and human studies become available, the clinical use of therapeutic resources will be based on scientific evidence rather than on mere empiricism.

## Conclusion

LLLT at 660 nm (15 mW, 7.5 J/cm^2^ )and 780 nm (70 mW, 3 J/cm^2^) might modulate the activation of J774 macrophages in a inflammatory condition simulation. Further studies are needed to elucidate the mechanisms that underlie such modulation as well as to assess the effects of irradiation on other relevant functions of macrophages.

## References

[B01] Tidball JG, Villalta SA (2010). Regulatory interactions between muscle and the immune
system during muscle regeneration. Am J Physiol Regul Integr Comp Physiol.

[B02] Wehling M, Spencer MJ, Tidball JG (2001). A nitric oxide synthase transgene ameliorates muscular
dystrophy in mdx mice. J Cell Biol.

[B03] Villalta SA, Nguyen HX, Deng B, Gotoh T, Tidball JG (2009). Shifts in macrophage phenotypes and macrophage
competition for arginine metabolism affect the severity of muscle pathology in
muscular dystrophy. Hum Mol Genet.

[B04] Schwab N, Waschbisch A, Wrobel B, Lochmüller H, Sommer C, Wiendl H (2008). Human myoblasts modulate the function of
antigen-presenting cells. J Neuroimmunol.

[B05] Nguyen HX, Tidball JG (2003). Interactions between neutrophils and macrophages promote
macrophage killing of rat muscle cells in vitro. J Physiol.

[B06] Gordon S (2003). Alternative activation of macrophages. Nat Rev Immunol.

[B07] Bibikova A, Oron U (1993). Promotion of muscle regeneration in the toad (Bufo
viridis) gastrocnemius muscle by low-energy laser irradiation. Anat Rec..

[B08] Bibikova A, Oron U (1995). Regeneration in denervated toad (Bufo viridis)
gastrocnemius muscle and the promotion of the process by low energy laser
irradiation. Anat Rec.

[B09] Oliveira NM, Parizzotto NA, Salvini TF (1999). GaAs (904-nm) laser radiation does not affect muscle
regeneration in mouse skeletal muscle. Lasers Surg Med.

[B10] Weiss N, Oron U (1992). Enhancement of muscle regeneration in the rat
gastrocnemius muscle by low energy laser irradiation. Anat Embryol (Berl).

[B11] Lopes-Martins RA, Marcos RL, Leonardo PS, Prianti AC Jr, Muscará MN, Aimbire F (2006). Effect of low-level laser (Ga-Al-As 655 nm)on skeletal
muscle fatigue induced by electrical stimulation in rats. J Appl Physiol.

[B12] De Almeida P, Lopes-Martins RÁ, Tomazoni SS, Silva JA Jr, De Carvalho PT, Bjordal JM (2011). Low-level laser therapy improves skeletal muscle
performance, decreases skeletal muscle damage and modulates mRNA expression of
COX-1 and COX-2 in a dose-dependent manner. Photochem Photobiol.

[B13] Young S, Bolton P, Dyson M, Harvey W, Diamantopoulos C (1989). Macrophage responsiveness to light
therapy. Lasers Surg Med.

[B14] Gavish L, Perez LS, Reissman P, Gertz SD (2008). Irradiation with 780 nm diode laser attenuates
inflammatory cytokines but upregulates nitric oxide in
lipopolysaccharide-stimulated macrophages: implications for the prevention of
aneurysm progression. Lasers Surg Med.

[B15] Mesquita-Ferrari RA, Ribeiro R, Souza NHC, Silva CAA, Martins MD, Bussadori SK (2011). No effect of low-level lasers on in vitro myoblast
culture. Indian J Exp Biol.

[B16] Da Silva TD, Mesquita-Ferrari RA, Souza NHC, Silva CAA, Martins MD, Bussadori SK (2010). Efeito da laserterapia de baixa potencia sobre a
proliferação de mioblastos C2C12. Fisioter Bras.

[B17] Fujihara NA, Hiraki KR, Marques MM (2006). Irradiation at 780 nm increases proliferation rate of
osteoblasts independently of dexamethasone presence. Lasers Surg Med.

[B18] Sousa LR, Cavalcanti BN, Marques MM (2009). Effect of laser phototherapy on the release of TNF-alpha
and MMP-1 by endodontic sealer-stimulated macrophages. Photomed Laser Surg.

[B19] Bolton PA, Young S, Dyson M (1990). Macrophage responsiveness to light therapy- a dose
response study. Tissue repair research Unit Division of anatomy.

[B20] Bolton P, Young S, Dyson M (1991). Macrophage responsiveness to light therapy with varying
Power and energy densities. Laser Ther.

[B21] Silva DF, Mesquita-Ferrari RA, Fernandes KP, Raele MP, Wetter NU, Deana AM (2012). Effective transmission of light for media culture,
plates and tubes. Photochem Photobiol.

[B22] Dourado DM, Favero S, Baranauskas V, Da Cruz-Hofling MA (2003). Effects of the Ga-As laser irradiation on myonecrosis
caused by Bothrops Moojeni snake venom. Lasers Surg Med.

[B23] Barbosa AM, Villaverde AB, Guimaraes-Souza L, Ribeiro W, Cogo JC, Zamuner SR (2008). Effect of low-level laser therapy in the inflammatory
response induced by Bothrops jararacussu snake venom. Toxicon.

[B24] Barbosa AM, Villaverde AB, Sousa LG, Munin E, Fernandez CM, Cogo JC (2009). Effect of low-level laser therapy in the myonecrosis
induced by Bothrops jararacussu snake venom. Photomed Laser Surg.

[B25] Mesquita-Ferrari RA, Martins MD, Silva JA Jr, Da Silva TD, Piovesan RF, Pavesi VC (2011). Effects of low-level laser therapy on expression of
TNF-α and TGF-β in skeletal muscle during the repair. Lasers Med Sci.

[B26] De Souza TO, Mesquita DA, Ferrari RA, Dos Santos Pinto D Jr, Correa L, Bussadori SK (2011). Phototherapy with low-level laser affects the remodeling
of types I and III collagen in skeletal muscle repair. Lasers Med Sci..

[B27] Baptista J, Martins MD, Pavesi VC, Bussadori SK, Fernandes KP, Dos Santos Pinto D Jr (2011). Influence of laser photobiomodulation on collagen IV
during skeletal muscle tissue remodeling after injury in rats. Photomed Laser Surg.

[B28] Fernandes KP, Alves AN, Nunes FD, Souza NH, Silva JA Jr, Bussadori SK (2013). Effect of photobiomodulation on expression of IL-1β in
skeletal muscle following acute injury. Lasers Med Sci.

[B29] Mehrsai A, Afsharpad M, Afsharpad M, Mohydin M, Ansari B, Pourmand G (2009). The effect of low-level helium-neon (HeNe) laser
radiation on the secretion of cytokines that promote chronic graft rejection - An
in vitro study. Med Laser App.

[B30] de Lima FM, Villaverde AB, Albertini R, De Oliveira AP, Faria HC No, Aimbire F (2010). Low-level laser therapy associated to N-acetylcysteine
lowers macrophage inflammatory protein-2 (MIP-2) mRNA expression and generation of
intracellular reactive oxygen species in alveolar macrophages. Photomed Laser Surg.

[B31] Dube A, Bansal H, Gupta PK (2003). Modulation of macrophage structure and function by low
level He-Ne laser irradiation. Photochem Photobiol Sci.

[B32] Emre Y, Nübel T (2010). Uncoupling protein UCP2: when mitochondrial activity
meets immunity. FEBS Lett.

[B33] Gerlier D, Thomasset N (1986). Use of MTT colorimetric assay to measure cell
activation. J Immunol Methods.

[B34] Chung H, Dai T, Sharma SK, Huang YY, Carroll JD, Hamblin MR (2012). The nuts and bolts of low-level laser (light)
therapy. Ann Biomed Eng.

[B35] Gao X, Xing D (2009). Molecular mechanisms of cell proliferation induced by
low power laser irradiation. J Biomed Sci.

